# Stop Using the Modified Work APGAR to Measure Job Satisfaction

**DOI:** 10.1155/2011/406235

**Published:** 2011-12-07

**Authors:** Thelma J. Mielenz, Robert F. DeVellis, Michele C. Battie, Timothy S. Carey

**Affiliations:** ^1^Department of Epidemiology, Mailman School of Public Health, Columbia University, 722 West 168th Street Room 809, New York, NY 10032, USA; ^2^Division of Physical Therapy, Department of Allied Health Sciences, School of Medicine and The Cecil G. Sheps Center for Health Services Research, The University of North Carolina at Chapel Hill, Chapel Hill, NC 27599, USA; ^3^Department of Health Behavior and Health Education, Gillings School of Global Public Health and the Thurston Arthritis Research Center, School of Medicine, The University of North Carolina at Chapel Hill, Chapel Hill, NC 27599, USA; ^4^Department of Physical Therapy, University of Alberta, Edmonton, AB, Canada T6G 2R3; ^5^Department of Medicine, School of Medicine and the Cecil G. Sheps Center for Health Services Research, The University of North Carolina at Chapel Hill, Chapel Hill, NC 27599, USA

## Abstract

*Background*. The psychometric properties of the Modified Work APGAR (MWA) scale are not established, yet researchers use this scale as an overall measure of job satisfaction. *Objective*. Perform psychometric analyses on the MWA scale using data from two populations. *Methods*. A landmark occupational cohort and a clinical cohort are populations with low back pain studied. The first five items of the MWA scale measure social support from coworkers, one item measures dissatisfaction with job tasks, and the sixth item measures lack of social support from a supervisor. Exploratory principal components analyses were conducted in both cohorts. *Results*. In both cohorts, the first five items of the MWA scale loaded consistently onto one factor, social support from coworkers subscale. *Conclusions*. Unless researchers are interested in measuring social support from coworkers only, future studies should use other reliable and valid instruments to measure a broad range of psychosocial work characteristics.

## 1. Introduction

 Approximately 50% of the US workforce has an episode of low back pain (LBP) each year [1, 2]. Researchers and clinicians recognize the multifactorial nature of LBP and its outcomes [2–5]. Over the last decade, studies have focused on not just the physical or medical factors of LBP in the workplace, but also the psychological and social environment at work [2, 5, 6].

 Clinicians screen for a myriad of different types of “flags” as outline in a 2011 publication by the “Decade of the Flags” Working Group, including (1) “red flags” for the possibility of serious spinal pathology; (2) “orange flags” for serious psychological symptoms; (3) “yellow flags” for pain beliefs, catastrophizing and coping skills; (4) “blue flags” for beliefs about work; (5) “black flags” for system problems (e.g., insurance) [2]. Research has established that both yellow and blue flags are associated with developing long-term disability from LBP [2, 5–7]. Clinicians screen for these flags during clinical examinations to identify “at risk” individuals who need strategies to manage their pain and keep active so they can return to normal activities [8].

 The quest for a psychometrically sound and efficient brief screening tools to identify “blue flags” in those patients or workers with LBP who are at risk for making the transition from acute to chronic LBP continues [2]. This study evaluates the psychometric properties using the Modified Work APGAR (MWA) scale, an instrument commonly used to measure “blue flags” or psychosocial work characteristics [6, 9–13]. The original Work APGAR is a five-item scale that focused on social support from coworkers in the workplace and was derived from the Family APGAR [9, 14, 15]. APGAR is an acronym for adaptation, partnership, growth, affection, and resolve [15]. The premise of the original Work APGAR is that social support from coworkers is similar to the social support of a family [9, 15]. To create the MWA, two items were added to the original Work APGAR, one about satisfaction with job tasks and the other about social support from a supervisor [9].

 The MWA measures perceived social support at work from coworkers and a supervisor as well as satisfaction with job tasks (Table 1). The MWA's greatest advantage is that it is brief [11]. If clinicians are expected to screen for these “blue flags,” then a very short measurement for unhappiness in the workplace would decrease the burden of this screening. However, a disadvantage of the MWA is that researchers continue to use the MWA but its psychometric properties are not well established [9–11]. There is no reported construct validation. Construct validation is the extent a proposed measure relates to other measures with similar theoretical bases [16–18]. Williams et al. attempted to validate the MWA using a principal components analysis but did not have a large enough sample for that analysis, with an *n* = 82 [6].

 The aim of this research is to examine the MWA scale, a commonly used measure of psychosocial work characteristics, using a classical test theory approach in subjects from two different populations, an occupational setting, and a clinical setting. Similar psychometric properties in these two populations separated in space and time would substantially strengthen the validation of the MWA. The specific hypotheses regarding the MWA scale are as follows.

1. The MWA has similar psychometric properties when used in a worker and a patient population.
2. The first five items ((a)–(e)) load on one construct but items (f) (I enjoy the tasks involved in my job) and (g) (I get along with my closest or immediate supervisor) are only moderately related to the construct.
3. The MWA is correlated more strongly with mental well-being than physical well-being.

## 2. Materials and Methods

This is a secondary analysis of two cohorts, a Worker cohort and a Patient cohort. The Worker cohort is a group of workers from the study entitled *Prospective study of work perceptions and psychosocial factors affecting the report of back injury *[9]. This landmark investigation is referred to as the “Boeing Study” [9]. The purpose of the Boeing study was to observe the association between back problems and physical, psychosocial, and workplace factors reported among workers in an industrial setting [9]. The study population was employees of the Boeing Company in Washington State who were free of LBP at baseline. Participation was open to 4027 employees receiving hourly wages. 75% (3,020) of those solicited volunteered. The 3020 volunteers first completed a cardiovascular-risk questionnaire and then underwent testing [9]. The baseline questionnaire collected psychosocial data using the Minnesota Multiphasic Personality Inventory (MMPI) and the MWA in addition to demographics and work history information. The 566 true-false questions on the MMPI were divided into ten clinical scales, three validity scales, and one scale of LBP [9].

 The Patient cohort is from the study entitled* Training primary care physicians to give limited manual therapy for low back pain *[13]. This was a randomized controlled trial of manual therapy (8 standardized maneuvers taught to primary care practitioners) versus “enhanced” care [13]. Thirty-three eligible physicians in North Carolina recruited 335 patients with acute LBP in this cohort. Patients met the following criteria: “age 21 to 65, acute LBP of less than 2 months' duration (acute or gradual onset), no prior spinal surgery or chymopapain therapy, no severe osteoarthritis or osteopenia, no pregnancy, no history of non-skin malignancy, no morbid obesity, no prior manual therapy by the enrolling physician, and no neurologic deficits detected in the physical examination [13].” Relevant covariates, including the MWA, were measured at baseline. The patients were reinterviewed at 2, 4, and 8 weeks after the baseline evaluation. The result was that the groups did not differ on the main outcomes [13].

### 2.1. Measures

 The MWA scale is shown in Table 1. Correlations were conducted on variables from the Worker cohort to determine if the MWA shares more variance (statistical variability) with mental well-being or with physical well-being [19]. Partialling the MWA in this manner was intended to gain insight into what factors the MWA measures and to help establish construct validation [16, 19]. Ideal mental and physical well-being scales were not available in the data, but mental well-being and physical well-being proxy scales were used instead. The conception of these proxy scales is explained below.

 The “mental well-being scale” was made up from the MMPI. The MMPI is commonly used to identify personality types but also has been used with LBP patients to predict chronicity from acute LBP [20–26]. The following clinical scales from the MMPI were used: 1(Hypochondriasis), 2(Depression), 3(Hysteria), 7(Anxiety), 9(Hypomania), and the LBP scale (Table 2). The LBP clinical scale was developed in the 1950s to differentiate between organic and nonorganic LBP [27].

 A “physical well-being scale” was created using relevant variables from the baseline clinical demographic characteristics listed in Table 2. The clinical variables chosen related to physical well-being as a consequence of LBP as well as to general health. No standardized physical functioning scales were available.

### 2.2. Data Analysis

Exploratory principal components factor analyses (EPCFAs) were conducted using the baseline MWA scores in the Worker and Patient cohorts to see if the factor structure was similar in these two populations. EPCFAs were conducted on the “mental well-being scale” and the “physical well-being scale” to clarify their factor structure and possibly form a multipoint scale to use in correlations. Cronbach's alpha reliability coefficient divides a scale with more than two response categories in two and gives the average of all the possible combinations in order to measure a scale's internal consistency among the items and was calculated after the EPCFAs for each factor present [19].

 A correlation matrix using Pearson's correlations was computed to assess relationships between the MWA and other variables in the Worker cohort. This matrix included MMPI clinical scales 1, 2, 3, and 7 from the mental well-being scales; three variables from the physical well-being scales: (1) recent LBP, (2) doctors seen for back pain, and (3) months on prescriptions for back pain in last 2 years.

 Guilford has suggested a minimum of 200 for sufficient power for factor analysis [28]. Both the Patient (*N* = 240) and Work cohorts (*N* = 1588) had more than 200 subjects. STATA was used for the analysis [29].

This study was submitted to the UNC Institutional Review Board and found exempt (ID 01-1480).

## 3. Results

 Of the 3020 volunteers for the Worker cohort study, 1588 subjects had complete MWA information. The Worker cohort was mostly white, male, middle-aged, high school educated, and married. Over a third of the cohort had a prior history of being treated for back injury and over a third smoked (Table 3).

 Of the 335 patients, recruited for the Patient cohort, forty patients refused and 295 were enrolled in the study. Two hundred forty patients provided complete MWA scales. All of these patients reported that they were employed in the last three months. A little less than half of the patients were male. The patients were mostly white, middle-aged, insured, and slightly more than half reported greater than a high school education. One-third of the patients smoke, and 80% had previous severe LBP episodes (Table 4).

**Figure 1:**
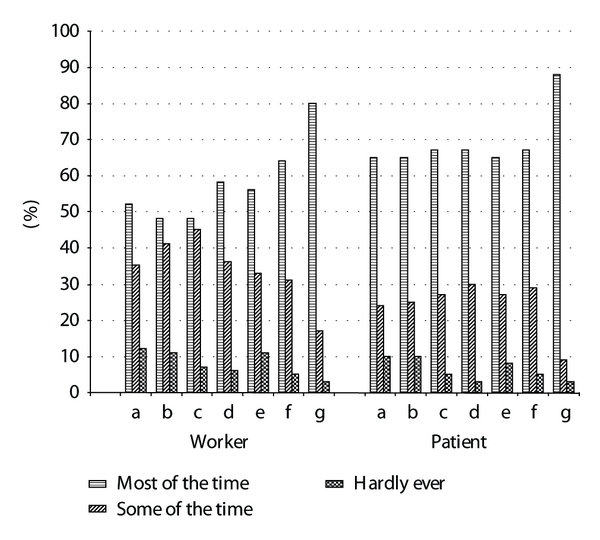
Comparison of the MWA responses in the Worker (*N* = 1588) and Patient (*N* = 240) cohorts.

Figure 1 depicts the percentage of subjects in each of the three response categories for the seven questions on the MWA ((a)–(g)) by study population. Visual inspection of this table reveals similar trends for the responses to the MWA scale across the two study populations. The Patient cohort appears to be slightly more satisfied in all of the MWA items (i.e., social support from coworkers and supervisor and job task satisfaction).

**Figure 2:**
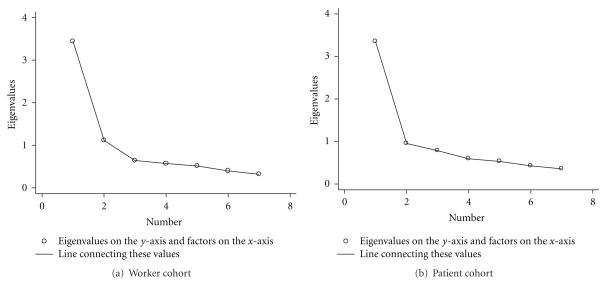
Scree tests for the Modified Work APGAR in each cohort.

The EPCFA of the MWA items for the Worker cohort identified two factors (2 eigenvalues >1) that explained 65% of the total variance among the items. Two factors were indicated by Cattell's scree test as well (Figure 2). Cattell's scree test is used to retain the first three factors before the slope levels off. After an oblique rotation which allows the factors to be correlated, the factor loadings made the interpretation of the two factors somewhat clearer. Thus, items (a)–(e) appear to have loaded on factor 1 and items (f) and (g) on factor 2. The interfactor correlations for the oblique factors were 0.35. Items (a)–(e) on the MWA had a Cronbach's alpha of 0.86. Since the second factor has an eigenvalue close to 1.00, an EPCFA was done to force one factor by raising the minimum value of the eigenvalue to be retained [29]. Items (a)–(e) all have loadings between  .76–.82. Items (f) and (g) have loadings  .48 and  .39, respectively. Items (a)–(g) had a Cronbach's alpha of 0.81.

 The EPCFA for the MWA scale on the Patient cohort loaded on one factor with an eigenvalue >1. This one factor determined 48% of the variance. Only one factor was present using the scree test as well (Figure 2). The factor loadings are moderate to high for items (a)–(e) (questions on social support from coworkers). Loadings for items (f) (job task satisfaction) and (g) (social support from supervisor) were moderate to low. Only one factor was retained; therefore, rotation was not necessary.

The second factor had an eigenvalue of 0.95 in the EPCFA of the Patient cohort MWA; therefore, two factors were forced to enhance the comparison with the Worker cohort by lowering the minimum value of the eigenvalue to be retained [29]. An oblique rotation was conducted to clarify the interpretation of the factors. Items (a)–(e) loaded moderately to highly on the first factor, and the loadings for items (f) and (g) were less than  .05. The opposite was true for the second factor with items (a)–(e) loadings no greater than 0.1 and items (f) and (g) with moderate to high loadings. The interfactor correlations for the oblique factors were 0.43. The seventh item MWA scale had a Cronbach's alpha of 0.81. Items (a)–(e) on the MWA had a Cronbach's alpha of 0.83.

**Figure 3:**
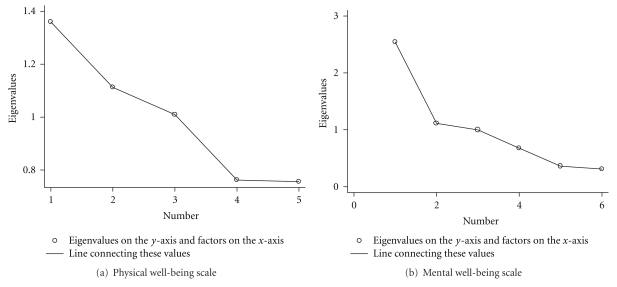
Scree tests for the well-being scales in the worker cohort.

 Exploratory principal components factor analyses (EPCFA) was done on the Worker cohort's “Physical Well-being Scale.” Two eigenvalues were >1, and the third was just over 1 (1.01). These three factors accounted for 70% of the total variance. A scree test also confirmed three factors (Figure 3). This was followed by an oblique rotation. The following three variables: (1) back pain on the day of the study participation or recent back pain causing work loss in the prior 6 months, (2) number of prescriptions for pain in 2 years, and (3) number of times patient has seen a doctor for back pain in last 2 years all had moderate loadings (range  .59–.74) on the first factor and represented the proxy “physical well-being” scale in the correlation matrix with the MWA.

Six of the MMPI clinical scales from the Worker cohort were analyzed by an EPCFA to see if the variables chosen loaded on a common factor. The *scree test* found two factors explaining 61% of the total variance (Figure 3). After an oblique rotation, the MMPI clinical scales 1 (Hypochondriasis), 2 (Depression), 3 (Hysteria), and 7 (Anxiety) had moderate to high loadings on factor 1 and were combined into a “Mental Well-being Scale” in the correlation matrix.

 The correlation matrix constructed from the Worker cohort showed an overall pattern of significant positive correlations between the MWA and mental well-being (Table 5). More specifically, the four combined MMPI scores were significantly correlated with the MWA. Two MMPI scales, the Scale 2 (Depression) and Scale 7 (Anxiety), correlated most highly with MWA. However, the relative magnitude of the correlations was low, with the highest correlation only being 0.2. Physical well-being did not correlate with the MWA.

## 4. Discussion

 Comparing the factor structure between these two populations, the MWA performed quite similarly. The factor structures of the MWA support the same conclusion: the additional 2 items that form the MWA (job task satisfaction and social support from supervisor) add little to the scale. The first 5 items of the MWA scale (the original Work APGAR scale which measures social support from coworkers) appeared to have construct validity in two very diverse populations.

This study afforded the opportunity to examine the psychometric properties of the MWA in two populations from different regions of the country, collected at different time periods (Worker cohort is from the early 1980s, and the Patient cohort is from the mid-1990s) and two different settings. Ideally, the MWA items should have a stable factor structure by looking at its characteristics in two quite different populations; this study was able to examine this issue. In these analyses, the MWA loaded on one factor in a population of patients in North Carolina seeking care for acute LBP and two factors in workers of the Boeing Company in Seattle, Washington.

This difference is more apparent than real. To make this point, two factors were forced for the Patient cohort. In both cohorts, one factor loaded on items (a)–(e) and one factor loaded on items (f) and (g). Although items (f) and (g) have high loadings on the second factor in both cohorts, it is only because they load differently from the items (a)–(e). Furthermore, coefficient alpha was attenuated by the inclusion of items (f) and (g) in both cohorts.

Comparing the factor structure between these two populations, the MWA performed quite similarly. In summary, the factor structures of the MWA support the same conclusion: the additional 2 items that form the MWA (job task satisfaction and social support from supervisor) add little to the scale. The first 5 items of the MWA scale (the original Work APGAR scale which measures social support from coworkers) appeared to have construct validity in two very diverse populations.

The MWA shared more variance with the “mental well-being scale” than with the “physical well-being scale” in the Worker cohort. Partialling the MWA in this manner was intended to gain insight into what factors the MWA measures and to help establish construct validity [16]. Williams et al. attempted to validate the MWA, but without adequate sample size (*n* = 82), and no other construct validity has been reported [6, 11].

 The descriptive statistics of the MWA for the Patient and Worker cohorts in this study are very similar to what has been reported by previous researchers. Williams et al. reported the following parameters for the MWA in their study: a mean of 9.6, a SD of 3.70, a range of 2–14, and a Cronbach's alpha of  .86 [6]. The MWA in the Patient cohort had a mean of 11.4, a SD of 3.70, range of 1–14, and a Cronbach's alpha of  .81. Because the Worker cohort used a scoring system that had lower scores representing more satisfaction, the MWA was adjusted by reverse scoring in the Worker cohort and had a mean of 10.5, a SD of 3.0, range of 7–21, and a Cronbach's alpha of  .82.

 The effect of setting could be a reason why the Patient cohort's MWA scores indicated more satisfaction. The Worker cohort was interviewed on the job. Although the investigators in both studies emphasized at the beginning of the baseline interview that all of the information would be kept confidential, the data collection was completely divorced from the occupational setting in the Patient cohort. The Patient cohort being slightly more satisfied contrasts with Volinn et al.'s claim that subjects may answer questions on job satisfaction more positively when the study is linked to the employer [30].

 There are methodological issues in sampling in both of the cohorts. The Worker cohort was made up of volunteer Boeing workers (~40% estimated participation rates), and no information was gathered on the nonvolunteers [30]. Information on the demographic and psychosocial characteristics of the nonresponders to the questionnaire was limited because many of these items were on the take-home questionnaire that nonresponders did not return [9, 30]. The physicians who recruited the Patient cohort were not randomly selected for this study, but they were community-based primary care physicians [13].

 The “physical well-being” proxies used in this study are not ideal. The variables chosen to represent the “physical well-being” scale did not all load on one factor. A standardized scale of physical well-being (e.g., SF-36) in subjects would have been preferable to the three variables used for the “physical well-being scale” in the correlation matrix [31].

## 5. Conclusion

We suggest using the original Work APGAR as a measure of coworker support, but stop using the MWA as an overall measure of psychosocial work characteristics. To measure additional psychosocial work characteristics or “blue flags,” other well-validated scales should be considered. A systematic review on the reliability and validity of instruments measuring job satisfaction found 7 quality instruments out of 29 retrieved [32]. One instrument was efficient, the five-item Andrew and Withey Job Satisfaction Questionnaire, and the rest had more than 18 items [32].

## Figures and Tables

**Table 1:** The Modified Work APGAR.

	Almost always	Some of the time	Hardly ever
(a) I am satisfied that I can turn to a fellow worker for help when something is troubling me.			

(b) I am satisfied with the way my fellow workers talk things over with me and share problems with me.			

(c) I am satisfied with the way my fellow workers accept and support my new ideas or thoughts.			

(d) I am satisfied with the way my fellow workers respond to my emotions, such as anger, sorrow, or laughter.			

(e) I am satisfied with the way my fellow workers and I share time together.			

(f) I enjoy the tasks involved in my job.			

(g) I get along with my closest or immediate supervisor.			

**Table 2:** The “Mental and Physical Well-being Variables” from the Worker cohort.

Mental well-being variables (clinical scales from the MMPI)
*1: Hypochondriasis or overly concerned with health
*2: Depression
*3: Hysteria
*7: Psychasthenia or anxiety
9: Hypomania or lower energy and activity level
LBP: Low back pain clinical scale

Physical well-being variables (physical variables from the Worker cohort)
*Back pain on the day of the study participation or recent back pain causing work loss in the prior 6 months
Excluded from exercise testing because of a history suggesting cardiovascular disease
Smokes
*Number of months using prescriptions for pain in last 2 years
*Number of doctors subject has seen for back pain in last 2 years

*Used as the mental and physical well-being scales in the correlation matrix.

**Table 3:** Worker cohort: clinical, work, and demographic characteristics at baseline *N* = 1588.

Characteristic	% or mean (SD)
White	95%
Married	80%
Male	74%
Years of education	12.5 (1.5)
Age	40 (11.3)
Prior workers' compensation claims	12%
Prior back surgery	2%
Number of months on prescriptions for pain in 2 years	1.1 (3.7)
Smokes	37%

Physical job demands of the subject's job reported at baseline	
(1) Light (mainly sedentary)	19%
(2) Medium-light	30%
(3) Medium (materials handling <50 pounds)	24%
(4) Medium-heavy	14%
(5) Heavy (regular lifting >50 pounds), sustained awkward work postures)	13%
Excluded from exercise testing because of a history suggesting cardiovascular disease	13%
Pain on SLR	2%
Back pain on the day of study participation or recent back pain causing work loss in the prior 6 months	13%
Back pain treated prior to 10 years ago	38%
Number of doctors a subject has seen for back pain in last 6 months	1.0 (1.6)
Number of doctors a subject has seen for back pain in last 2 years	1.1 (3.9)

MMPI Scales	
Hypochondriasis	53.8 (10.0)
Depression	55.4 (10.8)
Hysteria	55.6 (8,2)
Antisocial	56.0 (10.7)
Masculinity-femininity	55.9 (9.9)
Paranoia	54.1 (9.1)
Psychasthenia	54.1 (10.0)
Schizophrenia	54.7 (10.8)
Hypomania	55.2 (11.0)
Social introversion	53.7 (10.1)
Low back pain	53.2 (10.8)

**Table 4:** Patient cohort: clinical, work, and demographic characteristics at baseline *N* = 240.

Characteristic	% or mean (SD)
White	80%
Married	76%
Male	48%
Employed during past 3 months	100%
Health insurance	94%
Workers' compensation	17%
Age	41 (10)
Income >$20,000	75%
>High School	56%
Drinks	40%
Smokes	30%
Biomechanical demands	57%
Duration of LBP >2 weeks	27%
Sciatica: pain to knee or below	19%
Modified Roland scale	15.5 (5)

Number of severe LBP spells in lifetime	20% none
	52% 1–5
	28% >5

General health	
Excellent	19%
Very good	46%
Good	26%
Fair	7%
Poor	2%

**Table 5:** Correlation matrix for validity *N* = 1451.

		Modified Work APGAR Scale						
	Average (a)–(e)	(a)	(b)	(c)	(d)	(e)	(f)	(g)
“Mental Well-being Scale (MMPI Scales)”								
^†^Clinical scales:								
1	*0.057	0.030	0.038	*0.055	0.047	*0.057	0.016	−0.013
2	*0.20	*0.14	*0.1 8	*0.16	*0.150	*0.16	*0.17	*0.095
3	0.0033	−0.012	0.0045	0.0037	0.0001	0.017	*0.057	−0.013
7	*0.11	*0.069	*0.10	*0.077	*0.091	*0.11	*0.17	*0.083

Sum 1, 2, 3, and 7	*0.13	*0.079	*0.11	*0.10	*0.098	*0.12	*0.14	*0.054

“Physical Well-being Scale”								
Recent LBP	−0.0040	−0.0055	0.011	0.0092	−0.0028	−0.026	−0.0001	−0.031
Visits to MD for LBP last 2 years	0.014	0.0011	0.0089	0.028	−0.0044	0.023	0.045	−0.015
Prescriptions for pain in last 2 years	0.021	0.038	0.031	0.0042	0.0090	−0.0006	−0.015	0.0077

^†^Clinical scales on the MMPI: 1 = Hypochondriasis 2 = Depression 3 = Hysteria 7 = Anxiety.

*P value <.05.
